# Peak Identification in Evolutionary Multimodal Optimization: Model, Algorithms, and Metrics

**DOI:** 10.3390/biomimetics9100643

**Published:** 2024-10-19

**Authors:** Yu-Hui Zhang, Zi-Jia Wang

**Affiliations:** 1 School of Computer Science and Technology, Dongguan University of Technology, Dongguan 523808, China; yhzhang@dgut.edu.cn; 2 School of Computer Science and Cyber Engineering, Guangzhou University, Guangzhou 510006, China

**Keywords:** multimodal optimization, peak identification, evolutionary computation

## Abstract

In this paper, we present a two-phase multimodal optimization model designed to efficiently and accurately identify multiple optima. The first phase employs a population-based search algorithm to locate potential optima, while the second phase introduces a novel peak identification (PI) procedure to filter out non-optimal solutions, ensuring that each identified solution represents a distinct optimum. This approach not only enhances the effectiveness of multimodal optimization but also addresses the issue of redundant solutions prevalent in existing algorithms. We propose two PI algorithms: HVPI, which uses a hill–valley approach to distinguish between optima, without requiring prior knowledge of niche radii; and HVPIC, which integrates HVPI with bisecting K-means clustering to reduce the number of fitness evaluations (FEs). The performance of these algorithms was evaluated using the F-measure, a comprehensive metric that accounts for both the accuracy and redundancy in the solution set. Extensive experiments on a suite of benchmark functions and engineering problems demonstrated that our proposed algorithms achieved a high precision and recall, significantly outperforming traditional methods.

## 1. Introduction

Many real-world problems have multiple satisfactory solutions. When dealing with such multimodal problems, for two reasons, it is often desirable to locate multiple optima instead of a single optimum. First, multiple optima can provide useful domain knowledge of the problem at hand. Second, sometimes the optimal solution cannot be realized due to physical constraints. Under such circumstances, users can quickly switch to other solutions if multiple good solutions are provided. Multimodal optimization, which aims to find multiple optimal solutions to a given problem, has received increased attention recently.

A promising approach to multimodal optimization is bio-inspired optimization algorithms, which are population-based metaheuristics inspired by natural processes [1,2,3,4]. These algorithms have been successfully applied to various search and optimization tasks [5,6,7,8]. Evolutionary algorithms (EAs) represent a prominent branch of bio-inspired optimization algorithms [9,10]. Traditionally, the population of an EA will converge to a single solution, with the final output being the best solution found. Nevertheless, the intrinsic parallelism of EAs suggests that they should be able to simultaneously locate multiple optima for a multimodal problem. Over the years, a number of studies have been performed on the use of EAs to tackle multimodal optimization problems. The techniques developed are commonly referred to as “niching” [11,12], which preserve multiple stable niches and prevent global convergence to a single solution. Some prominent niching techniques include crowding [13,14], fitness sharing [15], restricted tournament selection [16], and species conserving [17]. Species are formed within basins of attraction using these niching techniques. According to whether the species are maintained explicitly, niching techniques can be divided into two categories, explicit niching and implicit niching techniques. Explicit niching techniques divide the population into subpopulations using radius-based methods [18,19], topology-based methods [20,21], or clustering methods [22,23]. In comparison, implicit niching uses a mechanism that facilitates the maintenance of population diversity. Species are formed automatically after several successive iterations using implicit niching techniques. Some recently proposed multimodal optimization algorithms [24,25,26] fall into this category.

**Figure 1:**
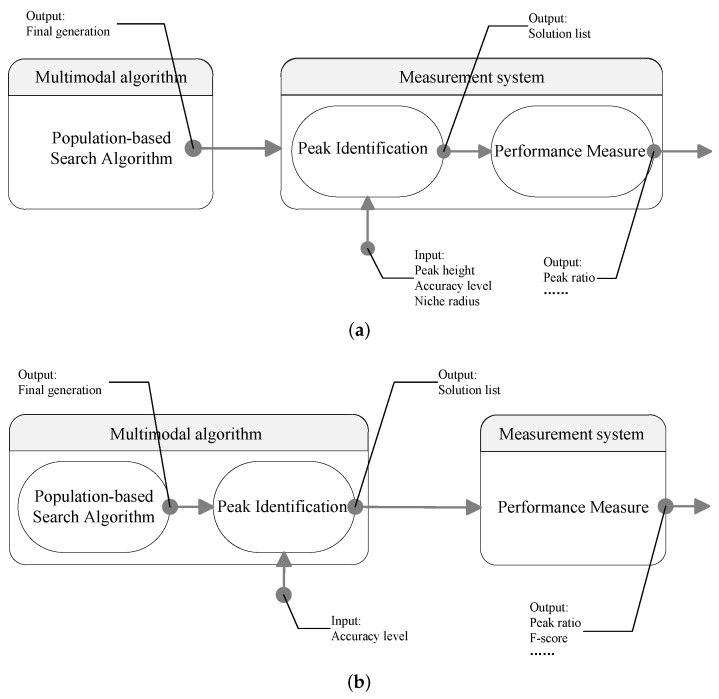
Frameworks of multimodal optimization: (**a**) current of multimodal optimization system, (**b**) modified multimodal optimization system.

The general framework of a current multimodal optimization system is shown in Figure 1a. It contains two parts. The first part is a multimodal optimization algorithm, and the second part is an evaluation system. In the first part, the output of an EA-based multimodal optimization algorithm is the final generation (or with an additional archive [27]). Note that there may be some duplicated or inferior individuals in the final generation. We need a procedure to extract the determined optima from the population. Therefore, in the evaluation system, efforts are first made to identify representative individuals. In the literature, the algorithm proposed by Parrott and Li [28,29] (denoted as PL hereafter) is commonly used to identify the found optima. It is the standard peak identification (PI) procedure for performance comparison of algorithms participating in a multimodal optimization competition. The input of the PL algorithm includes the final generation, the fitness value of global optima (peak height), a parameter called niche radius, and a user-specified accuracy level, while its output is a solution set containing all identified peaks.

There is a defect in the system model shown in Figure 1a. When dealing with real-world problems, to our knowledge, there does not exist a peak identification technique to extract the set of optima from the final generation. The aforementioned PL algorithm cannot be used, because it is impractical to know the niche radius and the peak height. For algorithms using explicit niching techniques, it is possible to output the best individual (species seed) in each species as the set of optima. However, if several species converge to the same peak, similar solutions will appear in the output. On the other hand, for implicit niching techniques, it is even harder to find representative individuals in a population. Existing algorithms generally use a population whose size is larger than the number of optima, and this potentially results in redundant solutions. If an algorithm outputs the final generation, users will have to try out each candidate solution (individual), which is a tedious task.

To summarize, for practical use, multimodal optimization algorithms should explicitly output the number of found optima and their positions. It should be the multimodal algorithm that engages peak identification, rather than the evaluation system, as shown in Figure 1b. In this work, we propose a generic two-phase framework for multimodal optimization algorithms. As illustrated in Figure 1b, the first phase is a population-based search algorithm (SA), which have been extensively studied in the literature. The second phase is a peak identification (PI) process that is used to rectify the output of the population-based search algorithms. We suggest deploying the PI process in the optimization component of a multimodal optimization system rather than in the evaluation component. Differently from existing works that focused on SA, this paper is dedicated to the second phase and aims to design a PI algorithm that can be used to identify representative individuals in the optimization component. To this end, we propose new PI algorithms that eliminate the need for any a priori knowledge. Instead of using the niche radius parameter, we check whether two individuals are in the same region of attraction by applying the hill–valley approach [30]. The use of the hill–valley approach involves fitness evaluations (FEs). To reduce the cost of the FEs, a bisecting clustering technique is embedded in the developed PI algorithm. The resulting algorithm is termed HVPIC (hill–valley-based peak identification algorithm using clustering). To the best of our knowledge, this work is the first attempt to tackle the peak identification task without using a priori knowledge.

To study the performance of two-phase multimodal algorithms, a performance index called the F-measure [31] was introduced. Using the F-measure, multimodal algorithms that find more optima and output fewer redundant individuals can receive higher scores. The F-measure is an improvement on the traditional performance measure peak ratio (PR) [29]. PR only takes into consideration the number of optima found. In comparison, the F-measure also takes into account the redundancy rate of the output. It encourages multimodal algorithms to output a redundancy-free solution list. Experiments were carried out on a number of widely used test functions to investigate the effect of the designed PI algorithm. The results showed that the HVPIC model was able to correctly identify the representative individuals.

There are some conventions used in this paper: the terms “peak” and “optimum” are used interchangeably when we are solving a maximization multimodal problem. In addition, we differentiate between the terms “niche” and “species” [32]. Each region of attraction is called a niche and each subpopulation is called a species.

The rest of this paper is organized as follows: Section 2 reviews the peak identification algorithms used in the literature. Section 3 details the proposed peak identification algorithm. A comprehensive performance measure for multimodal algorithms is introduced in Section 4. In Section 5, experiments were carried out to investigate the performance of the HVPIC model. Experimental results and discussions are also included in this section. Finally, conclusions are drawn in Section 6.

## 2. Niche Radius-Based Peak Identification

To evaluate the performance of a multimodal algorithm, it is necessary to determine the number of distinct peaks located by the population. The PL algorithm is an algorithm designed to handle this task. In this section, we first review the PL algorithm, then discuss defects in the algorithm.

### 2.1. PL Algorithm

**Figure 2:**
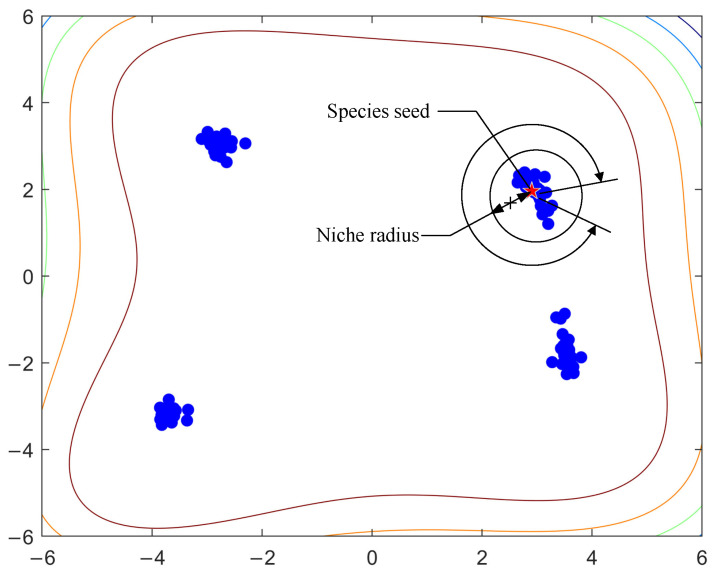
Illustration of the principle of the PL algorithm. The colored lines represent the contours of the Himmelblau function.

The general idea of the PL algorithm is as follows: We first identify the best individual in a species. Then, we delete all other individuals whose distance to the species seed is less than a threshold value *r* (a parameter called niche radius). The process is repeated until we obtain all the species seeds. Figure 2 illustrates the idea.

The pseudo code of the PL algorithm is shown in Algorithm 1. The algorithm maintains a solution list *S*, which is initially empty. The final generation is first sorted in descending order, according to their fitness values. Then, the algorithm looks through the individuals in the sorted list and adds an individual to the solution list if it satisfies the following two conditions:
**Algorithm 1** PL**Input:**      
$L_{sorted}$–individuals sorted in descending order;       *r*–niche radius;      
ε–accuracy level;      
ph–the fitness of global optima;
**Output:**
  *S*–a set of individuals identified as solutions
 1:S←∅ 2:**while** not reaching the end of $L_{sorted}$ **do** 3:   Get the next unprocessed $p\in L_{sorted}$; 4:   notNewNiche← False; 5:   **if** ph−fit(p)≤ε **then** 6:     **for** each s∈S **do** 7:        **if** ∥s−p∥≤r **then** 8:            notNewNiche← True; 9:            **break**;10:        **end if**11:     **end for**12:     **if** notNewNiche== False **then**13:        S←S∪{p};14:     **end if**15:   **end if**16:**end while**

1. The difference between the optimal fitness value and the fitness of the individual is less than the specified accuracy level ε;
2. The individual belongs to a different niche from those in *S*:
  (1) ∀s∈S: ∥s−X∥>r
  where ∥s−X∥ denotes the Euclidean distance between *s* and *X*.

The output of the PL algorithm is *S*, which contains all the distinct global optima (species seeds) found.

### 2.2. Complexity Analysis of PL Algorithm

The complexity of the PL algorithm can be measured by the number of distances calculated (i.e., the number of executions of the condition test in line 7). Suppose there are *N* individuals in the $L_{sorted}$. In the best case, all the individuals are in the same niche, and the solution list *S* will contain only one element throughout the running process. The code in the for loop (lines 7–10) will execute only once for each $p\in L_{sorted}$. Consequently, the running time of the PL algorithm, in the best case, is O(N). In the worst case, all the individuals have reached the accuracy level and each individual belongs to a different niche. For the *i*-th individual in $L_{sorted}$, i−1 comparisons are needed before it is added to *S*. Therefore, the complexity T(N) of the PL algorithm is bounded by the following inequality:

(2) $$T\left(N\right)\leq \sum_{i=1}^{N}\left(i-1\right)=\frac{N(N-1)}{2}$$

It can be inferred that the worst-case complexity of the PL algorithm is $O(N^{2})$. Generally, the total number of distance calculations will not exceed $N_{s}\cdot N$, where $N_{s}$ is the number of species in the final generation. This is a much tighter upper bound of the complexity of the algorithm, since $N_{s}$ is generally much less than *N*.

### 2.3. Difficulty of Setting the Niche Radius

If all species in the final generation have sufficiently converged, the identification task becomes trivial. As an extreme case, suppose that individuals in the same species have converged to a single point, then we just need to delete the duplicated individuals in the final generation and we will obtain the answer, i.e., each individual exclusively represents a possible optimum. However, for more general cases, due to the limited budget for fitness evaluations, it is more likely that individuals in the same species are scattered within a promising region.

The PL algorithm uses a parameter called niche radius to differentiate between individuals in different niches. Research works have been carried out on the setting of the niche radius. In [33], Deb and Goldberg proposed a simple method to set the niche radius. They first calculate the radius of the smallest hypersphere containing the feasible space, which is given as

(3) $$R=\frac{1}{2}\sqrt{\sum_{i=1}^{D}{\left(x_{i}^{u}-x_{i}^{l}\right)}^{2}}$$

where *D* represents the number of dimensions of the problem at hand, and $x_{i}^{u}$ and $x_{i}^{l}$ are the upper and lower bounds of the *i*-th dimension, respectively. Then, the niche radius is estimated as

(4) $$r=\frac{R}{\sqrt[{D}]{N_{g}}}$$

where $N_{g}$ is the number of global optima. The formula is based on the assumption that the optima are evenly distributed in the search space [34]. The main drawback of this approach is that it is practically impossible to know the number of optima in advance. Moreover, a fixed niche radius setting implicitly assumes equally sized and spherically shaped niches [35,36]. To tackle the problem of finding unevenly spread optima, Jelasity and Dombi [37] proposed using a radius function instead of a single radius. Some adaptation methods were later proposed in the literature [18,26]. Note that the adaptation of the niche radius is a long-term process and is difficult to generalize to other algorithms. The setting of the niche radius for the PL algorithm remains a difficult task.

**Figure 3:**
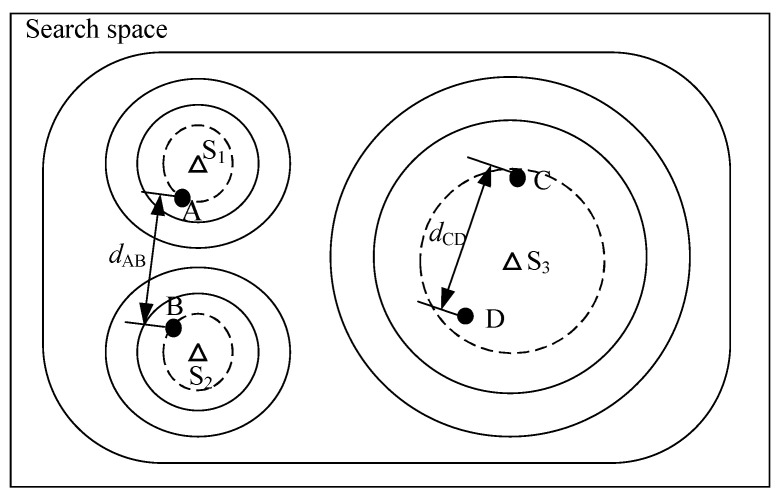
A situation where the PL algorithm fails.

Here, we illustrate the challenges in finding the right setting for the niche radius. As long as the niche radius is set to less than the distance between the two closest optima, individuals in two different niches can be well separated. On the other hand, if the niche radius is set too small, there is a danger that individuals in the same niche will be regarded as being in two species. This situation occurs when the regions of attraction are of different shapes or sizes. Figure 3 gives an example to illustrate the situation. In Figure 3, there are three niches and four individuals. The stars ($S_{1}$, $S_{2}$, and $S_{3}$) are used to denote the peaks and the solid points (A, B, C, and D) are used to represent the individuals. Two individuals are located in the niches on the left, and the other two are located in the niche on the right. Suppose that the distance between A and B is $d_{AB}$ and the distance between C and D is $d_{CD}$. We assume that the fitness values of the individuals have reached the required accuracy level. To separate A and B, the niche radius must be smaller than $d_{AB}$. On the other hand, to join C and D, the niche radius must be larger than $d_{CD}$. The fact that $d_{CD}$ is larger than $d_{AB}$ leads to a dilemma when setting the niche radius.

**Figure 4:**
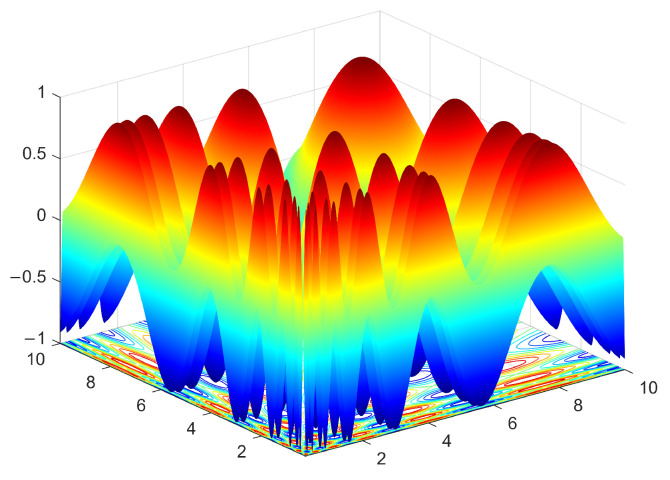
Landscape of the Vincent function.

**Figure 5:**
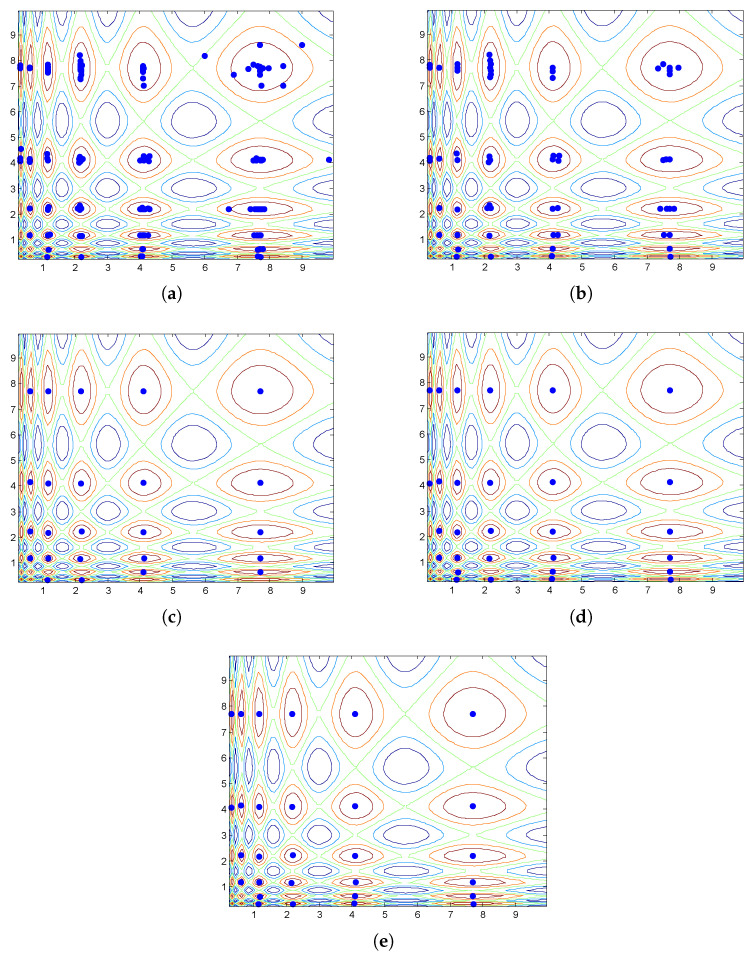
Effect of PL, HVPI, and HVPIC. The colored lines represent the contours of the function landscape. (**a**) Distribution of individuals in the final generation of the NCDE algorithm. (**b**) Optima identified by PL with r=0.1. (**c**) Optima identified by PL with r=0.5. (**d**) Optima identified by HVPI. (**e**) Optima identified by HVPIC.

The disadvantage of using niche radii is observed when we use NCDE [38], a state-of-the-art population-based search algorithm, to optimize the Vincent function. The Vincent function is a frequently used test function in the literature [29]. Figure 4 shows a landscape of its 2-D version. The niches are of elliptical shape. They have different eccentricities and their sizes vary significantly. The parameters of the NCDE algorithm are set according to [38] and the population size is fixed at 200. The NCDE algorithm terminates after 100 iterations. Figure 5a shows the distribution of individuals in the final generation of the NCDE algorithm. The PL algorithm is used to identify the representative individuals. Its input parameters are set as follows: ε=0.1, ph=1.0. Two niche radii, i.e., r=0.1 and r=0.5, are tested in the example. The outputs of the PL algorithm with the two settings are shown in Figure 5b and c respectively. From Figure 5b, it can be seen that dozens of optima are identified. However, several of the optima are actually in the same region of attraction. The reason for this is that the niche radius is too small for some niches. On the other hand, a larger niche radius may jeopardize the detection of optima located in narrow niches, as depicted in Figure 5c. From Figure 5c, it can be observed that some optima are mistakenly excluded from the output.

## 3. The Proposed Topology-Based Peak Identification Algorithm

The PL algorithm works well when the following two conditions are met: (1) all peaks are of similar shape and size, (2) the niche radius is correctly set. However, in practice, the first condition is not always satisfied and the niche radius is difficult to set. Therefore, a more rigorous algorithm whose performance is robust to the problem landscape is desired. We therefore propose using topology-based methods. In this way, the final generation can be correctly divided into species, regardless of the shapes and sizes of the niches. Specifically, we developed two improved algorithms, as follows:

1. HVPI: a hill–valley-based peak identification algorithm.
2. HVPIC: a hill–valley-based peak identification algorithm coupling with clustering.

### 3.1. Hill–Valley-Based Peak Identification (HVPI)

#### 3.1.1. The HVPI Algorithm

**Figure 6:**
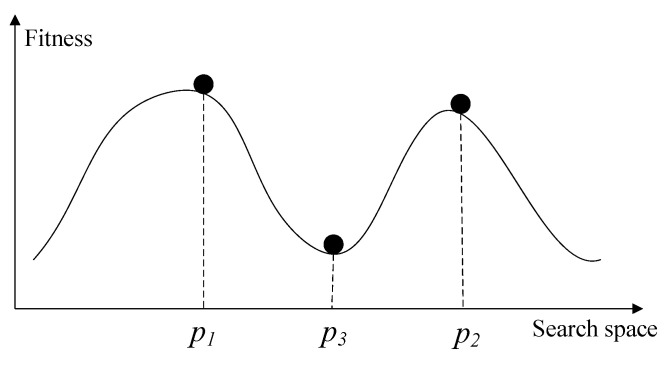
Illustration of hill–valley.

The first improvement is based on the intuition that two individuals are located in different niches if there is a valley between them. In the new HVPI algorithm, the topology-based hill–valley approach is used to determine whether two individuals are in the same niche. The hill–valley approach is rigorously defined in the following manner. For any two points $p_{1}$ and $p_{2}$ in the search space, a valley exists between them if there exists a point $p_{3}$ on the line segment *L* connecting $p_{1}$ and $p_{2}$ such that the fitness value at $p_{3}$ is less than the fitness values at both $p_{1}$ and $p_{2}$. Mathematically, the hill–valley condition can be expressed as

(5) $$fit\left(p_{3}\right)<min\left\{fit\left(p_{1}\right),fit\left(p_{2}\right)\right\}$$

where $p_{3}=p_{1}+\left(p_{2}-p_{1}\right)\cdot t$ and *t* is a real value within (0,1). To negate the existence of a valley, checking every point along the line segment *L* would be impractical. Therefore, the hill–valley approach employs an approximation scheme, where a decision is made based on a fixed number of sampled points. If the test returns TRUE, a valley is identified between the two individuals. Conversely, if the result is FALSE, it is likely that the two individuals belong to the same niche. The sample points are determined by an array $\mathrm{Samples}=[t_{1},t_{2},\ldots ,t_{\alpha }]$, where each element $t_{i}$ is within the interval (0,1) and α is the sample size. The detailed procedures are described in Algorithm 2 and Figure 6 illustrates the process.

The hill–valley approach has been adopted by researchers to enhance the niching performance of population-based multimodal algorithms [39,40,41]. In this paper, it is used as an elementary operation of the improved PI algorithm. By replacing the test condition in line 7 of Algorithm 1 with the hill–valley approach, we obtain a peak identification algorithm that can handle the situation in which niches have different shapes and sizes. In addition, the niche radius *r* is removed from the parameter list. The resulting algorithm is termed hill–valley-based peak identification (HVPI). For completeness, the pseudo code of the HVPI algorithm is provided in Algorithm 3.
**Algorithm 2** hill–valley**Input:**      $p_{1},p_{2}$–two individuals; **Output:**valleyExists–a Boolean value indicating whether there is a valley between the two individuals;1:valleyExists← False;2:**for** each t∈Samples (Samples is a fixed array of real values within [0, 1]) **do**3:   $p_{3}\leftarrow p_{1}+\left(p_{2}-p_{1}\right)\cdot t$;4:   **if** $\mathrm{fit}\left(p_{3}\right)<min\left\{\mathrm{fit}\left(p_{1}\right),\mathrm{fit}\left(p_{2}\right)\right\}$ **then**5:     valleyExists← True;6:     **break**;7:   **end if**8:**end for**

**Algorithm 3** HVPI**Input:**      
$L_{sorted}$–individuals sorted in decreasing fitness values;      
ε–accuracy level;      
ph–the fitness of global optima;
**Output:**
 *S*–a set of individuals identified as solutions
 1:S←∅ 2:**while** not reaching the end of $L_{sorted}$ **do** 3:   Get the next unprocessed $p\in L_{sorted}$; 4:   notNewNiche← False; 5:   **if** ph−fit(p)≤ε **then** 6:     **for** each s∈S **do** 7:        **if** hill-valley(*s*, *p*)==False **then** 8:            notNewNiche← True; 9:            **break**;10:        **end if**11:     **end for**12:     **if** notNewNiche== False **then**13:        S←S∪{p};14:     **end if**15:   **end if**16:**end while**


**Figure 7:**
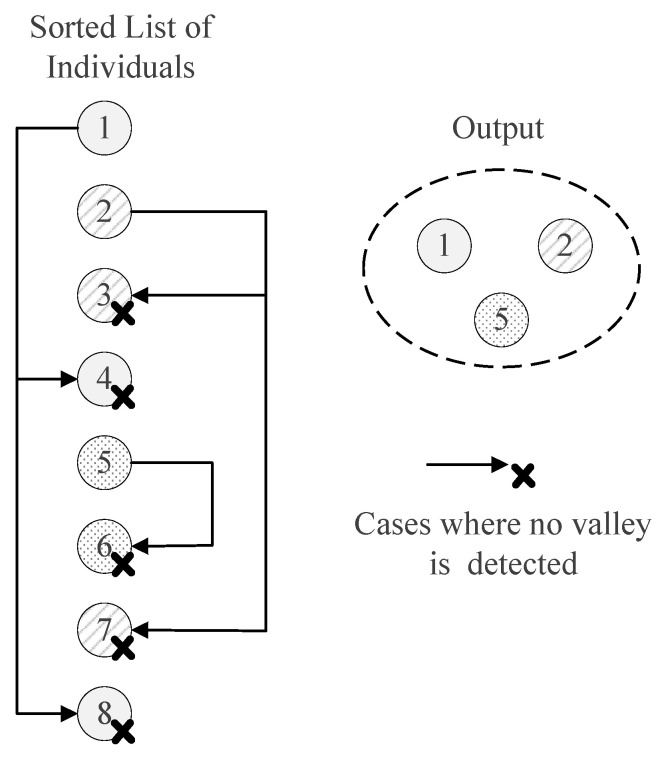
Illustration of HVPI.

To further aid comprehension, a diagram that visually represents the flow of operations within the HVPI algorithm is depicted in Figure 7. The algorithm accepts a list of individuals, $L_{sorted}$, arranged in order of their fitness values. It starts with the first individual, $X_{1}$, and subsequently evaluates the presence of a valley between $X_{1}$ and each subsequent individual in the list $\left\{X_{2},X_{3},\ldots ,X_{8}\right\}$ using the hill–valley approach. If no valley is found between $X_{1}$ and another individual, such as $X_{4}$, the algorithm concludes that these individuals belong to the same niche. As shown in the diagram, no valley is detected between $X_{1}$ and $X_{4}$, so $X_{4}$ is considered redundant and removed from $L_{sorted}$. The same logic applies to individual $X_{8}$. After the initial assessment, $X_{1}$ is extracted from $L_{sorted}$ and added to the solution set *S*. This procedure is repeated iteratively for the remaining individuals in $L_{sorted}$. In the end, the HVPI algorithm produces a solution set *S* that consists of non-redundant, optimally diverse individuals.

Continuing the experiment conducted at the end of Section 2, the HVPI algorithm is used to filter the final generation of the NCDE algorithm. The optima identified using HVPI are plotted in Figure 5d. It can be seen that no any two individuals in the output are located in the same niche.

#### 3.1.2. Discussion

In the optimization process, information about the problem is enclosed in a black box and is invisible to the PI algorithm. The distance between the two closest optima are unknown. Therefore, we developed a topology-based PI algorithm (HVPI) to avoid using this knowledge. While topology methods eliminate the need for fine-tuning the niche radius, they require sampling and evaluating new individuals to capture the landscape topology, incurring extra FEs. According to the complexity analysis of the PL algorithm, it can be inferred that, in the worst case, approximately $O(N_{s}\cdot N)$ FEs are required by HVPI. In the best case, approximately O(N) FEs are needed. In both cases, the number of FEs is related to the number of individuals *N*. In real-world applications where FEs are expensive or time-consuming, an excess requirement for FEs will reduce the practicability of the algorithm. Therefore, another design challenge for a topology-based PI algorithm is to reduce the number of FEs.

### 3.2. Hill–Valley-Based Peak Identification Using Clustering (HVPIC)

#### 3.2.1. Rationale

The second improved algorithm takes inspiration from clustering techniques. Clustering techniques have been adopted by researchers in the EC community to investigate population distributions. They can be used to discover the natural groupings of a set of individuals and thereby serve the purpose of dividing a population into multiple subpopulations [42,43].

Clustering techniques have become powerful tools for EAs in multimodal optimization. Generally, the clustering tendency of individuals is evident in the final stage of the optimization process and clustering algorithms are very effective when used in the stage. The rationale for the HVPIC algorithm is as follows. In HVPI algorithm, the hill–valley approach calls for a large number of pairs of individuals, without considering the population distribution. If a clustering algorithm is applied to the population, individuals assigned to a cluster are very likely to be in the same niche. This suggests that one can exploit the clustering outcome to avoid unnecessary use of the hill–valley approach, which will greatly reduce the number of FEs. Based on this consideration, a robust clustering algorithm named bisecting *K*-means algorithm is employed in HVPIC. According to the characteristic of the bisecting *K*-means algorithm, we show that the number of FEs consumed by HVPIC is proportional to the number of optima of the problem at hand.

#### 3.2.2. K-Means and Bisecting K-Means

Before moving on to the improved algorithm, a brief introduction to *K*-means and bisecting *K*-means is presented as a preliminary to the forthcoming discussions. *K*-means is one of the most popular clustering algorithms. Let $X_{1},\ldots ,X_{m}$ be a set of *m* points to be clustered into *K* clusters $C_{1},\ldots ,C_{K}$. Let $\mu_{i}$ be the centroid of cluster $C_{i}$. The objective of the *K*-means algorithm is to minimize the sum of the squared error (SSE):

(6) $$\mathrm{SSE}\left(\left\{C_{1},\ldots ,C_{K}\right\}\right)=\sum_{i=1}^{K}\sum_{X\in C_{i}}{∥X-\mu_{i}∥}^{2}$$

The detailed procedures of the algorithm can be found in [44]. *K*-means outputs a cluster list, which indicates the cluster assignments of the data points. Working in an iterative manner, the algorithm is, however, sensitive to the random initialization of cluster centroids.

Bisecting *K*-means, proposed by Steinbach et al. [45], is a straightforward extension of the basic *K*-means algorithm. It maintains a cluster list, which initially includes the cluster containing all the data points. In each iteration, the algorithm selects a cluster from the cluster list and splits the cluster into two smaller ones using the basic *K*-means algorithm. The two new clusters are then added to the cluster list. The process is repeated until the cluster list contains *K* clusters. Algorithm 4 shows the pseudo code of the bisecting *K*-means algorithm. Compared with *K*-means, the bisecting *K*-means algorithm is less susceptible to the initialization problem, because it tries several bisections and takes the one with the lowest *SSE*, and there are only two new centroids in each step [44].
**Algorithm 4** bisecting *K*-means**Input:**      
$X_{1},\ldots ,X_{m}$– a set of points to be clustered;       *K*–number of clusters;
**Output:**
  $L_{C}$– a cluster list indicating the cluster assignments of the points;
 1:Initialize a list of cluster $L_{C}$ to contain the cluster consisting of all the points; 2:**repeat** 3:   Remove the cluster *C* with the largest SSE from the list of clusters $L_{C}$; 4:   $\{C_{1},C_{2}\}\leftarrow$*K*-means(*C*,2) 5:   **for** i=2 to *number of trials* **do** 6:       $\{C_{1}^{\prime },C_{2}^{\prime }\}\leftarrow$*K*-means(*C*,2) 7:       **if** $\mathrm{SSE}\left(\left\{C_{1}^{\prime },C_{2}^{\prime }\right\}\right)<\mathrm{SSE}\left(\left\{C_{1},C_{2}\right\}\right)$ **then** 8:          $\left\{C_{1},C_{2}\right\}\leftarrow \left\{C_{1}^{\prime },C_{2}^{\prime }\right\}$ 9:     **end if**10:   **end for**11:   $L_{C}\leftarrow L_{C}\cup \left\{C_{1},C_{2}\right\}$12:**until** $|L_{C}|==K$ (the size of $L_{C}$ is equal to *K*)


#### 3.2.3. The HVPIC Algorithm

**Figure 8:**
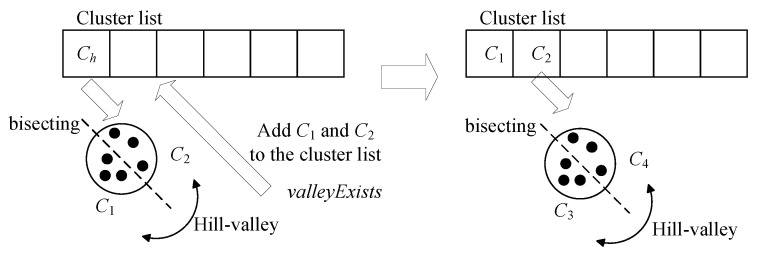
Illustration of the bisecting process of HVPIC.

The input of the HVPIC algorithm includes the final generation and a user-specified accuracy level. No problem-specific knowledge is required. The fitness value of the best individual is adopted as an estimated value of the peak height, as suggested in [34]. HVPIC maintains a solution list and a cluster list. The solution list is initially empty, whereas the cluster list contains a single cluster consisting of all the individuals in the population. In each iteration, the cluster $C_{h}$ at the head of the list is removed. Two new clusters ($C_{1}$ and $C_{2}$) are generated by applying *K*-means to $C_{h}$. Subsequently, the hill–valley approach is applied to check whether the representative individual (best individual) in $C_{1}$ lies in the same niche as the one in $C_{2}$. If the answer is yes, then it is considered that all the individuals in $C_{h}$ are in the same niche. Therefore, the best individual $cbest_{h}$ in $C_{h}$ is added to the solution list. Otherwise, $C_{1}$ and $C_{2}$ are added to the head of the cluster list for further division, as depicted in Figure 8. The above process is repeated until the cluster list is empty. Algorithm 5 gives the detailed procedures of HVPIC.
**Algorithm 5** HVPIC**Input:**      
POP–the final generation of an EA;       ε–accuracy level;
**Output:**
  *S*–a set of individuals identified as solutions;
 1:Initialize a cluster list $L_{C}$ to contain the cluster consisting of all the points, initialize the solution list; 2:ph=gbest.fitness //ph is the estimated peak height 3:**repeat** 4:   Remove the cluster $C_{h}$ at the head of the clusters list; 5:   **if** $|C_{h}|>1$ **then** 6:       $\{C_{1},C_{2}\}\leftarrow$*K*-means($C_{h}$,2); 7:       **for** i=2 to *number of trials* **do** 8:          $\{C_{1}^{\prime },C_{2}^{\prime }\}\leftarrow$*K*-means($C_{h}$,2); 9:          **if** $\mathrm{SSE}\left(\left\{C_{1}^{\prime },C_{2}^{\prime }\right\}\right)<\mathrm{SSE}\left(\left\{C_{1},C_{2}\right\}\right)$ **then**10:              $\left\{C_{1},C_{2}\right\}\leftarrow \left\{C_{1}^{\prime },C_{2}^{\prime }\right\}$11:        **end if**12:     **end for**13:   **else**14:     $cbest_{h}\leftarrow$ the best individual in $C_{h}$;15:     $S\leftarrow S\cup \{cbest_{h}\}$;16:     **continue**;17:   **end if**18:   $cbest_{1}\leftarrow$ the best individual in $C_{1}$;19:   $cbest_{2}\leftarrow$ the best individual in $C_{2}$;20:   **if** hill-valley($cbest_{1}$, $cbest_{2}$)==TRUE **then**21:     **if** $ph-cbest_{1}.\mathrm{fitness}\leq \varepsilon$ **then**22:        $L_{C}\leftarrow L_{C}\cup \left\{C_{1}\right\}$;23:     **end if**24:     **if** $ph-cbest_{2}.\mathrm{fitness}\leq \varepsilon$ **then**25:        $L_{C}\leftarrow L_{C}\cup \left\{C_{2}\right\}$;26:     **end if**27:   **else**28:     $S\leftarrow S\cup \{cbest_{h}\}$;29:   **end if**30:**until **$L_{C}=\emptyset$


In Algorithm 5, gbest denotes the best individual in the population. The number of trials in the bisecting process is set to 20. HVPIC inherits the robustness of the bisecting *K*-means algorithm. Moreover, some modifications have been introduced to make the algorithm suitable for the peak identification task.

1. The selection of the cluster to be bisected is simplified. In bisecting *K*-means, the cluster is chosen using specific rules (choose the largest cluster or the one with the largest SSE). In the HVPIC algorithm, the cluster at the head of the list is chosen.
2. The rule that determines whether new clusters should be added to the cluster list is redesigned. In bisecting *K*-means, two new clusters are added to the cluster list. In HVPIC, only clusters consisting of individuals from different niches are added to the cluster list.
3. The termination criterion of HVPIC also differs from bisecting *K*-means. Bisecting *K*-means terminates when there are *K* clusters in the cluster list. In comparison, HVPIC terminates when the cluster list is empty. The number of executions of the do-while block depends on the distribution of the population and the landscape of the problem at hand. This eliminates the need for specifying the number of clusters (species).

**Figure 9:**
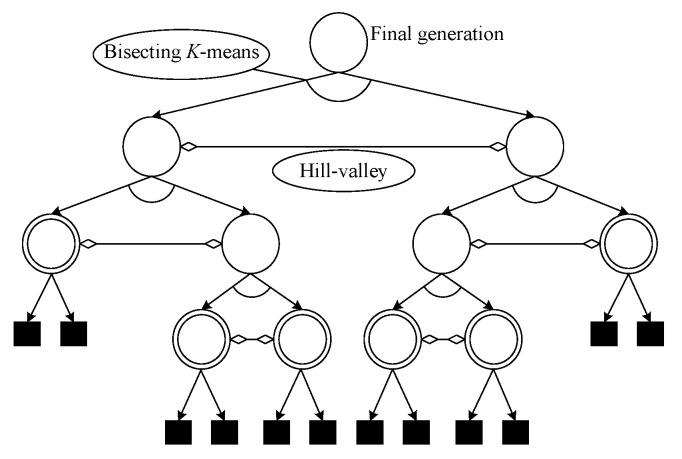
Tree representation of the bisecting process of HVPIC.

It is convenient to use a binary tree diagram to show the bisection process. Figure 9 illustrates the HVPIC algorithm. The root of the binary tree represents the cluster consisting of all the individuals. Arrows pointing from a parent node to its child nodes denote the bisection of a cluster. Each horizontal double-headed arrow indicates a call of the hill–valley approach. A node stops spanning when (1) the best individual in it does not reach the accuracy level or (2) the best individual in it belongs to the same niche as that in its sibling node. The nodes that stop spanning because of the first condition contribute nothing to the final solution set. The more general case is that leaf nodes (denoted by square in Figure 9) are formed due to the second condition. We use T to denote the final tree, and T’ is the tree generated by removing the leaf nodes of T. The leaf nodes of T’ (denoted by concentric circles) represent the clusters whose best individual is added to the solution list. Therefore, the number of leaf nodes of T’ is equal to the number of optima identified by HVPIC.

#### 3.2.4. Analysis of the Number of FEs Required by HVPIC

To analyze the number of FEs required by the HVPIC algorithm, we introduce the concept of full binary trees and one of its important properties.

**Definition** **1.** *A full binary tree is a tree in which every node other than the leaves has two children.*

**Property** **1.** *For any non-empty full binary tree with $N_{0}$ leaf nodes and $N_{1}$ internal nodes, $N_{0}=N_{1}+1$.*

The property can be easily proved by induction. According to the definition, $T^{\prime }$ is a full binary tree. Suppose the number of leaf nodes and the number of internal nodes of $T^{\prime }$ are $N_{0}$ and $N_{1}$, respectively. To generate T’ from the root, $N_{e}$ executions of the bisection process are conducted. Each execution will produce two new nodes. Therefore, we obtain the following equation:

(7) $$N_{0}+N_{1}-1=2N_{e}$$

By applying the property, we have $N_{e}=N_{0}-1$. Combining the result with $N_{0}=N_{i}$, it gives $N_{e}=N_{i}-1$, where $N_{i}$ is the number of optima identified by the HVPIC algorithm. Meanwhile, it can be noticed that the number of executions of the bisection process is equal to the number of calls of the hill–valley approach. The required number of FEs is therefore given by $O(\alpha N_{i})$, which is invariant of the population size. This is an important property. Generally, a large population size is required for problems with many optima. This property guarantees that the number of FEs required by HVPIC will not be influenced by the population size.

## 4. Performance Measure

Several performance measures have been proposed to evaluate the performance of EAs for multimodal optimization, among which the peak ratio (PR) and success rate (SR) are two frequently used measures [29]. Peak ratio is the percentage of successfully located peaks:

(8) $$PR=\frac{\sum_{i=1}^{NR}NPF_{i}}{N_{g}\cdot NR}$$

where $NPF_{i}$ denotes the number of global optima found in the *i*-th run, $N_{g}$ the number of global optima, and NR the number of runs. Success rate is the percentage of runs in which all the peaks are successfully located:

(9) $$SR=\frac{NSR}{NR}$$

where NSR is the number of successful runs.

The two performance measures are indifferent to the redundancy in the output. However, in some real-world applications (e.g., multi-object detection [46]), a redundant solution will result in a false-positive error. It is hard for users to remove redundant solutions manually. Therefore, a performance measure that forces multimodal algorithms to provide a redundancy-free output is desired. To this end, the concept of F-measure [31] is introduced. F-measure is an extensively used measure in the research area of information retrieval. It is a combination of two important concepts, i.e., precision and recall. Before introducing the F-measure, we first give the definitions of precision and recall in the context of multimodal optimization.

**Figure 10:**
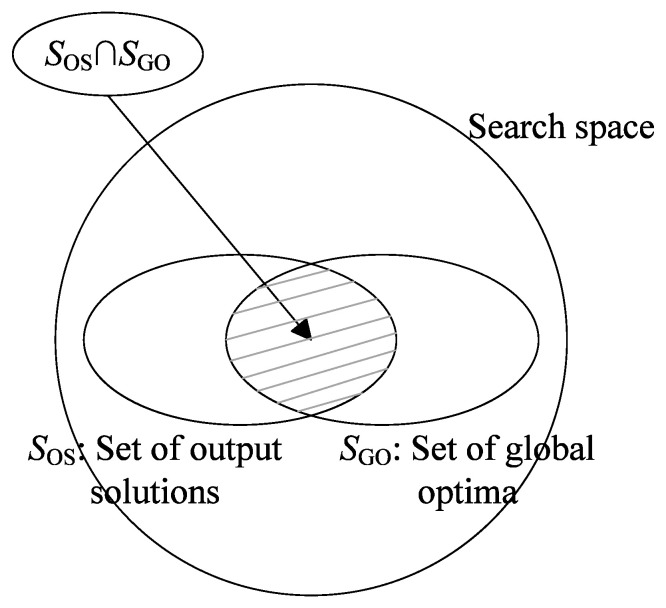
Illustration of precision and recall.

Consider a multimodal algorithm consisting of two components: a population-based search algorithm and a peak identification algorithm. At the termination of the search algorithm, we obtain a population of individuals. Then, we apply the PI algorithm to the population and obtain a set of output solutions (denoted as $S_{\mathrm{OS}}$). Suppose the set of global optima is given by $S_{\mathrm{GO}}$ (illustrated in Figure 10). Let |S| be the number of elements in set *S*. The precision and recall of a multimodal algorithm are defined as follows:

**Definition** **2.** *Precision is the fraction of actual optima which have been identified:*

(10) $$\mathrm{Precision}=\frac{|S_{\mathrm{OS}}\cap S_{\mathrm{GO}}|}{|S_{\mathrm{OS}}|}$$

**Definition** **3.** *Recall is the fraction of identified optima which are actual optima:*

(11) $$\mathrm{Recall}=\frac{|S_{\mathrm{OS}}\cap S_{\mathrm{GO}}|}{|S_{\mathrm{GO}}|}$$

The F-measure is the harmonic mean of the precision and recall. It provides a way to combine precision and recall into a single value. It is computed as follows:

(12) $$F=\frac{2\cdot \mathrm{Precision}\cdot \mathrm{Recall}}{\mathrm{Precision}+\mathrm{Recall}}$$

The F-measure assumes a high value only when both the recall and precision are high. In particular, *F* becomes 1 when all the optima have been located and all the redundant individuals have been removed. Maximizing *F* can be interpreted as an attempt to find the best compromise between precision and recall.

From the above definitions, it can be noticed that PR is equal to the average recall over multiple runs. Compared with PR, F-measure is a more comprehensive measure, since it takes into consideration both the peak ratio and the redundancy rate. If a multimodal algorithm outputs an entire population, it is probable that it will obtain a very low precision, resulting in low F-scores. Hence, an additional benefit of using the F-measure is that it encourages multimodal algorithms to remove redundant solutions before the output process. It is coupled with the new model (Figure 1b) to increase the practicability of evolutionary multimodal algorithms.

## 5. Experiments

To see whether the proposed PI algorithm can effectively remove the redundant solutions in the population, in this section, experiments were carried out on a set of benchmark functions. The performance of the algorithms was evaluated using the performance measure introduced in Section 4.

### 5.1. Experimental Setup

#### 5.1.1. Benchmark Functions

The CEC2013 benchmark function set [29] was adopted to study the performance of the PI algorithms. The function set contains 20 functions with different characteristics and levels of difficulty. They are listed in Table 1. All the functions are maximization problems. $F_{1}-F_{5}$ are simple, non-scalable, low-dimensional multimodal functions. $F_{6}-F_{12}$ are scalable multimodal functions. For $F_{6}$ and $F_{7}$, the number of global optima was determined by the dimensionality. For $F_{8}-F_{12}$, the number of global optima was independent of the dimensionality. $F_{9}-F_{12}$ are non-symmetric composition functions constructed using several basic functions. $F_{9}$ and $F_{10}$ are separable, while $F_{11}$ and $F_{12}$ are non-separable. More detailed descriptions about the test functions can be found in [29].

#### 5.1.2. Population-Based Search Algorithms and Parameter Settings

The proposed PI algorithm can be integrated with different population-based search algorithms. In the experiment, two popular population-based search algorithms, i.e., the neighborhood-mutation-based crowding DE (NCDE) [38] and the locally informed particle swarm (LIPS) [47], were adopted. The scale factor *F* and crossover rate Cr of NCDE were set to 0.5 and 0.1, respectively. The neighborhood size nsize introduced by LIPS was dynamically increased from 2 to 5 over the function evaluations. The settings of the population size and MaxFEs for the test functions are shown in Table 2. The final generation of each search algorithm was stored in an archive for further analysis.

Four PI algorithms (PI_0_, PL, HVPI, and HVPIC) were used to remove redundant solutions in the final generation, and the F-measure was adopted to evaluate the performance of the integrated algorithms (SA+PI). PI_0_ is a dummy PI algorithm that directly outputs the final generation. The niche radius required by PL was estimated using (3) and (4). Since the peak height (ph) was unavailable in the optimization process, the fitness value of the best individual in the final generation was used as an approximation. For topology-based PI algorithms, we assigned the sample array as Samples=[0.02,0.25,0.5,0.75,0.98]. The accuracy level ε used to determine whether an optimum was found was set to 0.01. The effect of ε will be investigated later in this section.

### 5.2. Overall Performance

**Figure 11:**
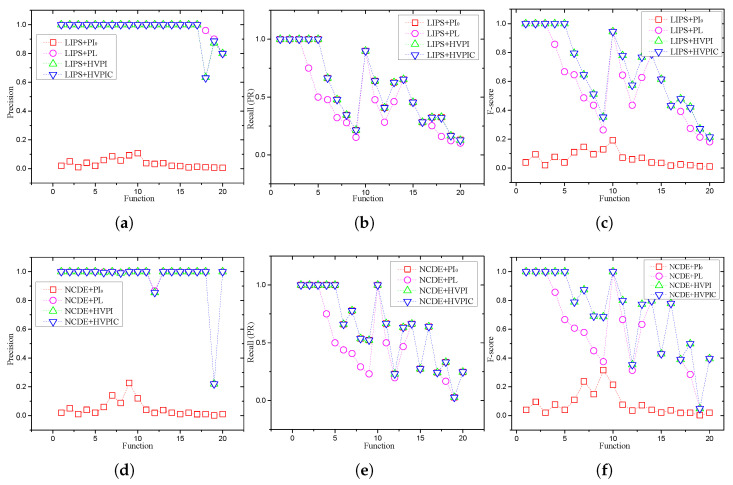
Precision, recall, and F-score of the integrated algorithms (SA+PI) on the benchmark functions. (**a**) Precision of LIPS+PI, (**b**) recall of LIPS+PI, (**c**) F-score of LIPS+PI, (**d**) precision of NCDE+PI, (**e**) recall of NCDE+PI, (**f**) F-score of NCDE+PI.

There were eight combinations when given two search algorithm (NCDE and LIPS) and four PI algorithms (PI_0_, PL, and HVPI, and HVPIC). The experimental results of the combinations are visualized in Figure 11. Figure 11 displays the average performance of each algorithm over 50 independent runs on the CEC2013 benchmark functions. This average was calculated to ensure statistical robustness, providing a reliable comparison of the peak detection capabilities of the HVPI, HVPIC, and PL algorithms. This approach reduced the potential impact of variations due to random initialization or other stochastic factors inherent in evolutionary algorithms.

In most cases, there was no significant difference between the HVPI and HVPIC algorithms in terms of the *F* value. For low dimensional functions with a small number of optima ($F_{1}-F_{5}$), the algorithms reached an *F* of 1. The FEs consumed by the HVPI and HVPIC algorithms in these cases were very small (reported in Table 3). For functions with many optima ($F_{6}-F_{7}$), only part of the optima were located by LIPS and NCDE. The recall values with these test functions were not as good as those for $F_{1}-F_{5}$, causing a drop in the *F* values. Moreover, an increase in the required number of FEs was observed as the number of optima in the final generation increased, as shown in Table 3. For complex composition functions ($F_{8}-F_{12}$), the *F* value oscillated with the change in the recall value. Note that the recall value was mostly determined by the search algorithm (i.e., NCDE and LIPS). The oscillation could not be eliminated using any PI algorithms. Therefore, the goal of the PI algorithms in these cases was to achieve a 100% precision, while preserving the maximum recall. The maximum recall is always given by PI_0_, since it does not remove any individuals in the population. From Figure 11, it can be seen that the recall obtained by HVPI and HVPIC was the same as that of PI_0_, indicating that HVPI and HVPIC were capable of preserving the maximum recall.

To evaluate the performance of the HVPIC algorithm and assess whether any peaks were missed due to the reduced calculations, we conducted an additional experiment on the Vincent function, and the results are shown in Figure 5e. For the specific case shown in Figure 5a, the output of HVPIC was identical to that of HVPI. To further investigate whether the reduction in FEs in HVPIC resulted in missed detections, Table 4 presents the average precision, recall, and *F* values for both the HVPI and HVPIC algorithms across all benchmark problems. The table shows that the performance of HVPIC was comparable to that of HVPI. While HVPIC significantly reduced the FEs compared to HVPI, it maintained a high peak detection accuracy, with no notable difference in the number of identified peaks across all tested multimodal landscapes. These results demonstrate that the HVPIC algorithm struck an effective balance between computational efficiency and accuracy.

**Figure 12:**
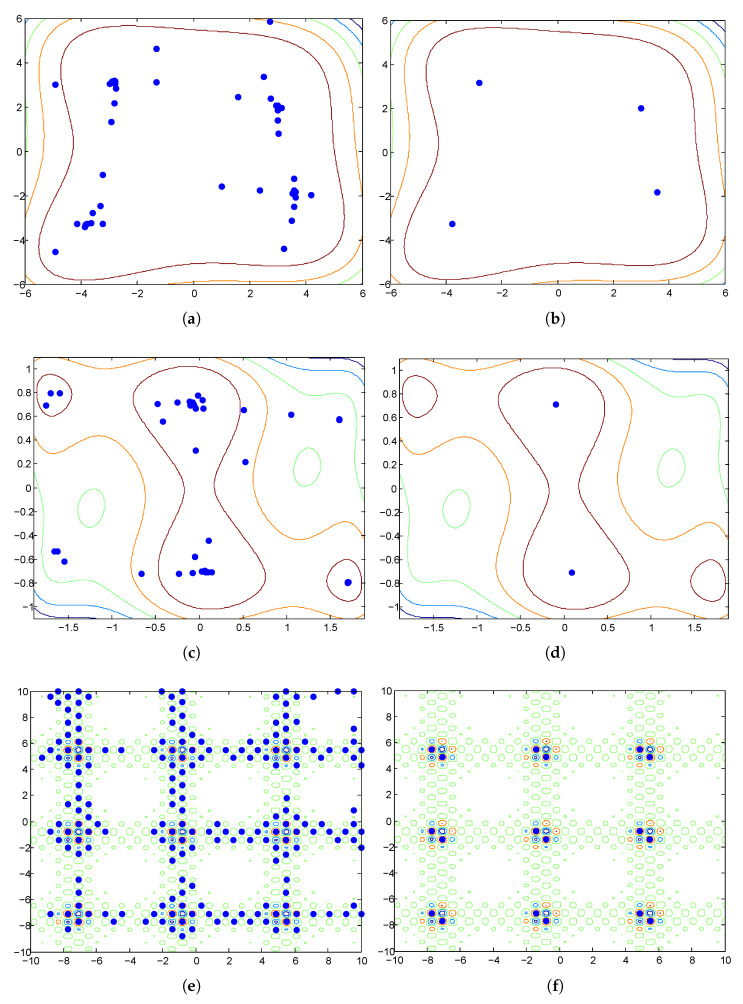

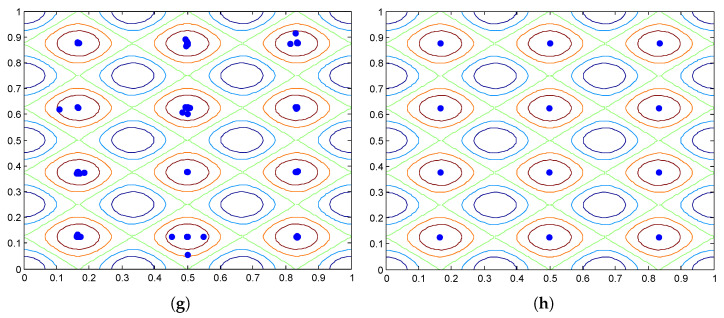
Effect of the HVPIC. The colored lines represent the contours of the test function. (**a**,**c**,**e**,**g**): Distribution of individuals in the final generation of NCDE on $F_{4}$(2D), $F_{5}$(2D), $F_{6}$(2D), and $F_{8}$(2D). (**b**,**d**,**f**,**h**): Optima identified by HVPIC on $F_{4}$(2D), $F_{5}$(2D), $F_{6}$(2D), and $F_{8}$(2D).

To see the effect of the peak identification algorithm, Figure 12 plots the population distribution of NCDE on two-dimensional test functions, as well as the optima identified by HVPIC. The *F* value of PL follows a similar pattern to that of HVPI and HVPIC. It can be noticed the recall value of PL was not as good as PI_0_, HVPI, and HVPIC, implying that some of the optima in the population were mistakenly removed. PL was able to catch up with HVPI and HVPIC when the estimated niche radius fit the landscape of the test function, but in other cases, the *F* value obtained by PL was unsatisfactory. In comparison, PI_0_ always obtained the highest recall among the PI algorithms. However, due to the existence of a large number of redundant individuals, its precision remained at a very low level.

It is worth noting that the experiments were conducted using particle distributions stored in a data file. These distributions were derived from the final iterations of LIPS and NCDE across 50 independent runs. The same particle distributions were fed into the four peak recognition algorithms, to ensure fair comparisons. To assess the robustness of the proposed algorithms, Table 5, Table 6, Table 7 and Table 8 present the best, worst, average, and error values for the precision and recall over 50 independent runs. The error values, in particular, indicate the robustness of the proposed HVPI and HVPIC algorithms. As shown in the tables, the precision values for PL, HVPI, and HVPIC were very similar in most test cases, with consistently low error values. In terms of recall, HVPI and HVPIC exhibited lower error values compared to PL, demonstrating an advantage in eliminating redundancy. This suggests that the redundant elimination capability of PL was not as effective as that of HVPI and HVPIC. Overall, the experimental results showed that both HVPI and HVPIC performed consistently well, with minimal variation in precision and recall, highlighting their robustness to variations in particle distributions.

### 5.3. Effect of Population Size

To investigate the influence of the population size parameter, different population sizes (50, 100, 200, 500, 800, and 1000) were adopted by LIPS. Experiments were conducted on $F_{6}$(2D), $F_{6}$(3D), $F_{7}$(2D), and $F_{7}$(3D). From Table 1, it can be seen that these test functions had a large number of optima. In particular, $F_{7}$(3D) had a number of global optima up to 216. The size of $S_{\mathrm{OS}}$ in these test cases varied significantly with respect to the setting of the population size.

**Figure 13:**
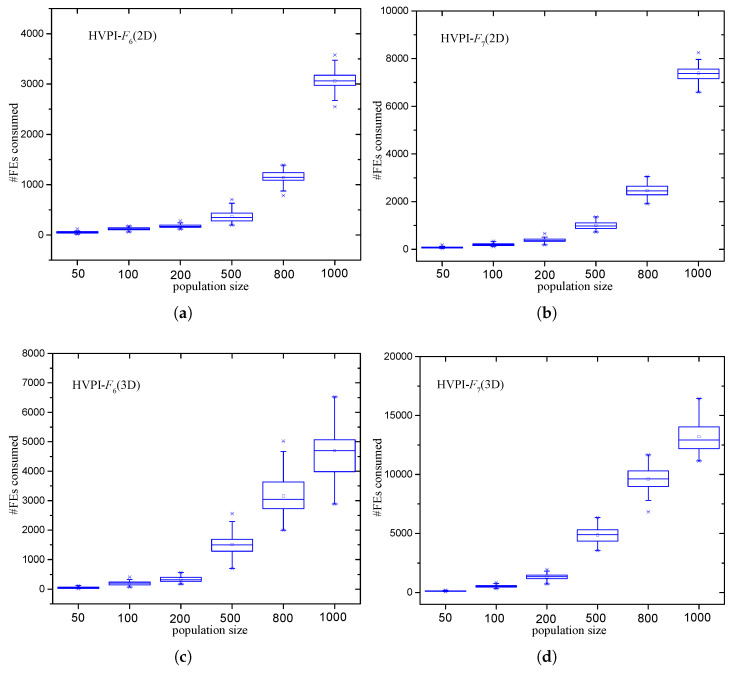

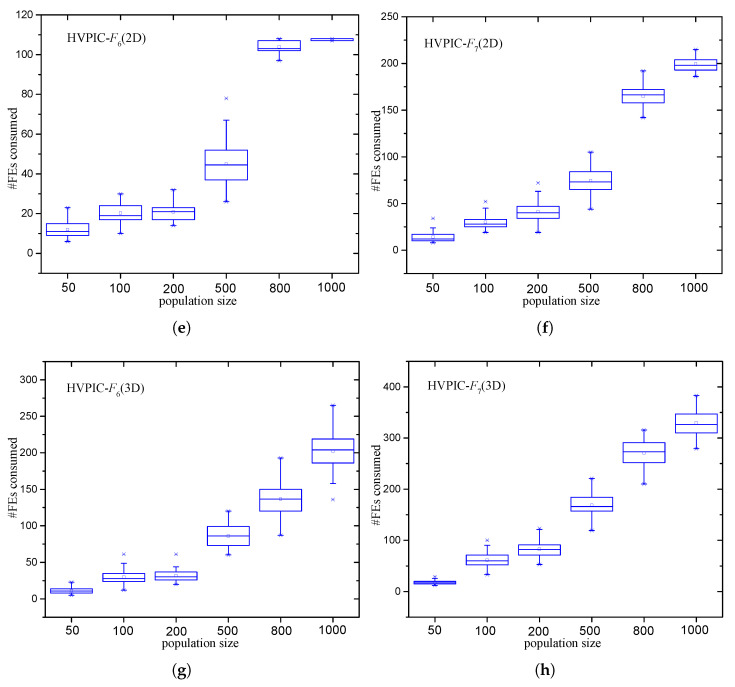
Number of FEs consumed when different population sizes were adopted. (**a**) HVPI-$F_{6}$(2D) (**b**) HVPI-$F_{7}$(2D) (**c**) HVPI-$F_{6}$(3D) (**d**) HVPI-$F_{7}$(3D) (**e**) HVPIC-$F_{6}$(2D) (**f**) HVPIC-$F_{7}$(2D) (**g**) HVPIC-$F_{6}$(3D) (**h**) HVPIC-$F_{7}$(3D).

**Figure 14:**
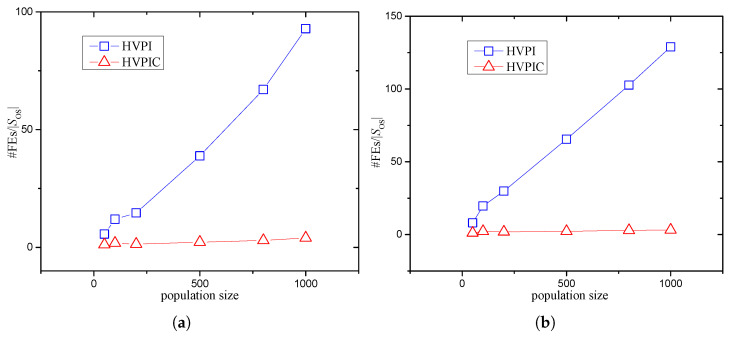
Number of consumed FEs per optimum versus population size. (**a**) $F_{6}$(3D) (**b**) $F_{7}$(3D).

The *F* values obtained by HVPI and HVPIC were the same. To study the influence of population size on the required number of FEs, boxplots are depicted in Figure 13. It can be observed that the numbers of FEs consumed by HVPI increased more dramatically than that of HVPIC. According to the analysis in Section 3, the number of FEs consumed by HVPIC was invariant of the population size. The increase in the number of FEs was mostly due to the growth in the size of $S_{\mathrm{OS}}$ (i.e., the number of identified optima). To illustrate this, #FEs$/|S_{\mathrm{OS}}|$ versus the population size is plotted in Figure 14 (#FEs denotes the number of FEs). In Figure 14, the polyline representing HVPIC is near horizontal, implying that the quantity #FEs$/|S_{\mathrm{OS}}|$ was not influenced by the population size. In contrast, for HVPI, #FEs$/|S_{\mathrm{OS}}|$ increased linearly as the population size grew.

### 5.4. Effect of Convergence Degree

In this section, we investigate the performance of the PI algorithms when the individuals in the population are not sufficiently converged. In general, the difficulty level of the peak identification task is associated with the degree of convergence of individuals. The more scattered the individuals, the more difficult the task. To generate populations that had different degrees of convergence, the termination criterion of LIPS was modified. Specifically, MaxFEs was set to 10,000, 25,000, 50,000, and 100,000, respectively. The experiment was conducted on $F_{7}$(3D) and the population size was fixed at 500.

The experimental results are listed in Table 9. The best results are displayed in boldface. None of the PI algorithms could reach 100% precision when MaxFEs was set to 10,000. This was due to the fact that the difference between the fitness of the best individual gbest and the exact peak height was not negligible. If the fitness of gbest is used as the estimated peak height, some imperfect individuals will also be identified as optima, causing a decline in precision.

**Figure 15:**
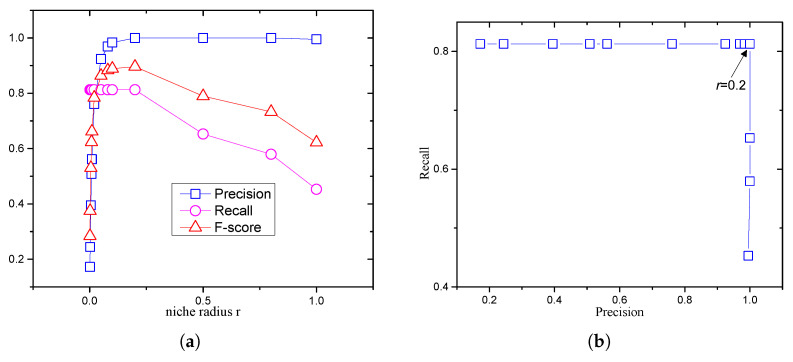
Performance of LIPS+PL on $F_{7}$(2D) using different niche radii. popsize=100, ε=0.01. (**a**) Trend in precision and recall. (**b**) Recall versus precision.

The HVPI and HVPIC algorithms reached the maximum *F* value when the MaxFEs were set to 25,000, 50,000, and 100,000. They had a consistent performance with different degrees of convergence. The results obtained by PL were worse than that of HVPI and HVPIC. The low recall values of PL were probably due to incorrect setting of the niche radius. Figure 15 shows the effect of the niche radius *r*. The use of a large niche radius tends to clear more redundant individuals in the population, so the precision increases as the niche radius increases. However, there is a danger that it will also eliminate possible optima near a species seed. Hence, the recall will correspondingly drop as the niche radius grows, as illustrated in Figure 15a. The recall versus precision is plotted in Figure 15b. There was only one setting of niche radius that gave the best compromise between precision and recall. It can be seen that the precision decreased rapidly if the niche radius was set to less than 0.2. Conversely, the recall dropped significantly if the niche radius was greater than 0.2. This indicates the difficulty in choosing a suitable niche radius.

### 5.5. Effect of Accuracy Level

The number of identified optima is related to the setting of the user-specified accuracy level ε. To investigate the influence of ε, the PI algorithms were tested at four levels of accuracy (0.1, 0.01, 0.001, and 0.0001). The population size and MaxFEs were fixed at 500 and 50,000, respectively.

The experimental results are tabulated in Table 10. The HVPI and HVPIC algorithms were able to reach the maximum recall value with respect to the different settings of ε. Meanwhile, they also achieved a precision value of 1, implying that their output solutions were redundancy-free. In comparison, PL failed to obtain a high recall with all settings of ε, indicating that some optima were mistakenly excluded.

To summarize, the HVPI algorithm was capable of reaching high *F* values under different parameter settings, but it required a large number of FEs. The HVPIC algorithm exhibited a similar performance as HVPI, and the number of FEs consumed was relatively small. In comparison, the performance of PL was very sensitive to the setting of the niche radius.

### 5.6. Application to Engineering Problems

To demonstrate the practical value of the HVPI and HVPIC algorithms in real-world engineering applications, we applied them to the multiple steady states problem, the molecular conformation problem, and the robot kinematics problem. A brief introduction to these problems is provided below.

- Multiple Steady States Problem: Evaluating multiple steady states in reaction networks is crucial in various chemical engineering applications, particularly in the analysis and design of chemical reactors. Steady states refer to conditions where the reaction rates and physical properties remain constant over time. Multiple steady states can exist in complex reaction networks, meaning there are several sets of conditions that satisfy the system’s governing equations.
- Molecular Conformation Problem: In molecular biology and drug design, determining the three-dimensional structure of a molecule is critical, particularly when identifying the minimum energy state or low-energy states. These low-energy conformations are likely the natural shapes of the molecule, significantly influencing its chemical reactivity, physical properties, and biological activity.
- Robot Kinematics Problem: A fundamental problem in robotics, kinematics studies the relationship between a robot’s joint configuration and the resulting motion of its end-effector. Understanding kinematics is crucial in determining the position, orientation, and velocity of robot components, without considering the forces driving the motion.

**Figure 16:**
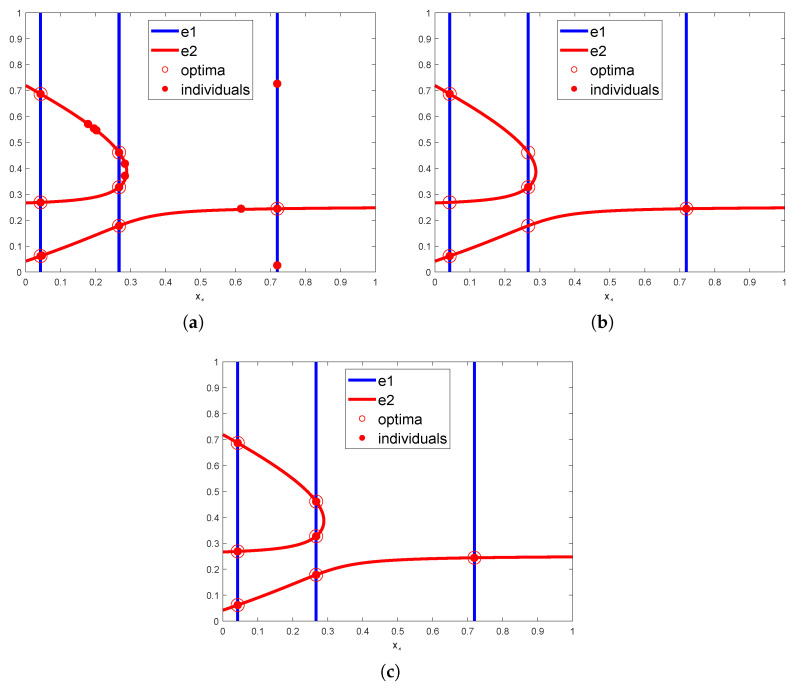
Experimental results on the multiple steady states problem. (**a**) Final generation of NCDE. (**b**) Output of PL. (**c**) Output of HVPIC.

These problems can be framed as multimodal optimization challenges. The mathematical formulations of the engineering problems can be found in [48,49,50]. We used NCDE to solve them and applied peak recognition algorithms to filter out redundant solutions. Table 11 summarizes the average optimization results, demonstrating that both HVPI and HVPIC identified optimal configurations with high accuracy. The three engineering problems are referred to as P1, P2, and P3, respectively. The proposed algorithms consistently outperformed the baseline PL algorithm, which is highly sensitive to parameter selection. Without prior knowledge, it can be difficult to determine an appropriate niche radius for PL, and an unsuitable choice can cause the F-value to drop significantly. To visually represent the effect of the proposed algorithms, Figure 16a shows the final generation of the NCDE algorithm, where the red circles denote optimal solutions and the red dots represent individuals in the final generation. e1 and e2 are the mathematical constraints of the problem. Figure 16b,c show the outputs of PL and HVPIC, respectively. The output of HVPI was identical to that of HVPIC. HVPIC correctly eliminated all redundant solutions, while PL mistakenly removed some optima, due to incorrect niche radius settings.

These results demonstrate the effectiveness of the proposed algorithms and their applicability to practical engineering challenges. Their ability to efficiently identify multiple optimal solutions in complex, multimodal landscapes suggests that they could be valuable in fields such as mechanical design, aerodynamics, and control system optimization. Future work will explore additional engineering applications, further validating the broad utility of the proposed algorithms.

### 5.7. Embedding HVPI and HVPIC into Group-Based Optimization Algorithms

While group-based optimization algorithms, such as PSO and DE, often rely on a single globally optimal particle or solution, this can limit their ability to explore multiple peaks in multimodal problems. In this regard, the integration of HVPI and HVPIC algorithms into such optimization methods becomes essential for overcoming this limitation. It is possible to integrate HVPI and HVPIC directly into the optimization process, enabling group-based algorithms to recognize and maintain multiple peaks during the search process. This can be achieved by incorporating a niche-based peak recognition mechanism that operates in parallel with the primary search algorithm. By doing so, the population is encouraged to explore multiple promising areas of the search space, rather than solely converging towards a single global solution. This approach enhances the exploration–exploitation balance of the algorithm and helps maintain diversity in the population, ensuring that multiple peaks are identified and explored during the optimization process.

## 6. Conclusions

In this paper, an attempt was made to rectify the output of multimodal optimization algorithms. The main results of the paper are summarized as follows:

1. We proposed a practical two-phase multimodal optimization model. The first phase is the population-based search algorithm that has been extensively studied in the literature. The second phase is the peak identification (PI) procedure. The new model containing PI eliminates the users’ burden of dealing with redundant solutions.
2. New PI algorithms that alleviate the need for problem-specific knowledge were developed. Specifically, a PI algorithm previously used in the evaluation system was integrated with the hill–valley approach, to avoid having to preset the niche radius. Furthermore, to reduce the number of FEs required by the hill–valley approach, we combined HVPI with bisecting *K*-means in the HVPIC algorithm. Theoretical analysis showed that the number of FEs consumed by the HVPIC algorithm was proportional to the number of identified optima.
3. To evaluate the performance of multimodal algorithms, the F-measure, which considers both precision and recall values, was introduced. Compared to the PR and SR measures that are widely used in the literature, the F-measure is more comprehensive, since it is capable of evaluating the redundancy rate of the outputs of multimodal algorithms.

Experiments were carried out to investigate the performance of the proposed PI algorithm. The experimental results showed that the HVPIC algorithm was able to correctly identify the representative individuals under different parameter settings. In most of the test cases, HVPIC reached the maximum recall and achieved precision of 100%. Meanwhile, the number of FEs used for the identification task was relatively small.

It is noteworthy that the recall is largely determined by the population-based search algorithm. The goal of PI is to eliminate redundant individuals in the final generation, to increase the precision while at the same time preserving the recall. When given a search algorithm, the maximum *F* value we can obtain is upper bounded by a certain value. Therefore, more effective search algorithms are of great importance for improving the overall performance of multimodal algorithms on complex problems (e.g., $F_{12}$(10D) and $F_{12}$(20D)). In future work, we plan to implement and test the effectiveness of embedding HVPI and HVPIC in a range of optimization algorithms to validate the broader applicability and impact of the proposed methods.

## Figures and Tables

**Table 1:** Test Functions.

No.	Function	Name	ph	$N_{g}$
1	$F_{1}$(1D)	Five-Uneven-Peak Trap	200	2
2	$F_{2}$(1D)	Equal Maxima	1	5
3	$F_{3}$(1D)	Uneven Decreasing Maxima	1	1
4	$F_{4}$(2D)	Himmelblau	200	4
5	$F_{5}$(2D)	Six-hump Camel Back	1.03163	2
6	$F_{6}$(2D)	Shubert	186.7309	18
7	$F_{7}$(2D)	Vincent	1	36
8	$F_{6}$(3D)	Shubert	2709.0935	81
9	$F_{7}$(3D)	Vincent	1	216
10	$F_{8}$(2D)	Modified Rastrigin	−2	12
11	$F_{9}$(2D)	Composition Function 1	0	6
12	$F_{10}$(2D)	Composition Function 2	0	8
13	$F_{11}$(2D)	Composition Function 3	0	6
14	$F_{11}$(3D)	Composition Function 3	0	6
15	$F_{12}$(3D)	Composition Function 4	0	8
16	$F_{11}$(5D)	Composition Function 3	0	6
17	$F_{12}$(5D)	Composition Function 4	0	8
18	$F_{11}$(10D)	Composition Function 3	0	6
19	$F_{12}$(10D)	Composition Function 4	0	8
20	$F_{12}$(20D)	Composition Function 4	0	8

**Table 2:** Settings of the Popsize and MaxFEs for the test functions.

Function	Popsize	MaxFEs
$F_{1}$ to $F_{5}$ (1D or 2D)	100	1.00 × 10^4^
$F_{6}$ to $F_{7}$ (2D)	200	2.00 × 10^4^
$F_{8}$ to $F_{11}$ (2D)	100	5.00 × 10^4^
$F_{6}$ to $F_{7}$ (3D)	500	5.00 × 10^4^
$F_{8}$ to $F_{12}$ (3D or higher)	200	1.00 × 10^5^

**Table 3:** Average number of FEs used in the peak identification process.

Function	NCDE+HVPI	NCDE+HVPIC	LIPS+HVPI	LIPS+HVPIC
$F_{1}$(1D)	1.00	1.00	1.00	1.00
$F_{2}$(1D)	475.12	29.94	220.00	30.18
$F_{3}$(1D)	52.10	4.90	42.90	5.00
$F_{4}$(2D)	266.70	23.00	590.44	23.00
$F_{5}$(2D)	210.90	11.00	378.00	11.00
$F_{6}$(2D)	74.20	11.36	104.50	17.88
$F_{7}$(2D)	1827.04	120.92	1800.94	94.42
$F_{6}$(3D)	1008.60	43.22	440.52	31.94
$F_{7}$(3D)	13,443.90	379.76	9368.30	254.48
$F_{8}$(2D)	323.52	47.44	65.56	10.48
$F_{9}$(2D)	73.14	14.70	7.32	3.54
$F_{10}$(2D)	8.14	2.96	4.90	2.66
$F_{11}$(2D)	76.12	22.48	5.28	2.76
$F_{11}$(3D)	206.00	15.20	5.76	2.92
$F_{12}$(3D)	98.32	11.70	6.20	2.84
$F_{11}$(5D)	326.34	21.74	2.08	1.30
$F_{12}$(5D)	173.48	10.64	4.24	2.20
$F_{11}$(10D)	170.84	10.90	10.30	5.74
$F_{12}$(10D)	5.80	1.70	19.62	5.58
$F_{12}$(20D)	289.34	10.88	88.20	7.46

**Table 4:** Average precision, recall, and F values obtained by HVPI and HVPIC on benchmark problems.

Function	HVPI			HVPIC		
	***Precision***	***Recall***	***F***	***Precision***	***Recall***	***F***
$F_{1}$(1D)	1.00	1.00	1.00	1.00	1.00	1.00
$F_{2}$(1D)	1.00	1.00	1.00	1.00	1.00	1.00
$F_{3}$(1D)	1.00	1.00	1.00	1.00	1.00	1.00
$F_{4}$(2D)	1.00	1.00	1.00	1.00	1.00	1.00
$F_{5}$(2D)	1.00	1.00	1.00	1.00	1.00	1.00
$F_{6}$(2D)	0.99	0.66	0.79	0.99	0.66	0.79
$F_{7}$(2D)	1.00	0.78	0.87	1.00	0.78	0.87
$F_{6}$(3D)	0.99	0.54	0.69	0.99	0.54	0.69
$F_{7}$(3D)	1.00	0.52	0.69	1.00	0.52	0.69
$F_{8}$(2D)	1.00	1.00	1.00	1.00	1.00	1.00
$F_{9}$(2D)	1.00	0.67	0.80	1.00	0.67	0.80
$F_{10}$(2D)	0.86	0.23	0.35	0.86	0.23	0.35
$F_{11}$(2D)	1.00	0.63	0.77	1.00	0.63	0.77
$F_{11}$(3D)	1.00	0.66	0.80	1.00	0.66	0.80
$F_{12}$(3D)	1.00	0.28	0.43	1.00	0.28	0.43
$F_{11}$(5D)	1.00	0.64	0.78	1.00	0.64	0.78
$F_{12}$(5D)	1.00	0.24	0.39	1.00	0.24	0.39
$F_{11}$(10D)	1.00	0.33	0.50	1.00	0.33	0.50
$F_{12}$(10D)	0.22	0.03	0.05	0.22	0.03	0.05
$F_{12}$(20D)	1.00	0.25	0.40	1.00	0.25	0.40

**Table 5:** Best, worst, average, and standard deviation of precision across 50 independent runs of NCDE.

Function	PI				HVPI				HVPIC			
	***Best***	***Worst***	***Avg.***	***Std.***	***Best***	***Worst***	***Avg.***	***Std.***	***Best***	***Worst***	***Avg.***	***Std.***
$F_{1}$(1D)	1.00	1.00	1.00	0.00	1.00	1.00	1.00	0.00	1.00	1.00	1.00	0.00
$F_{2}$(1D)	1.00	1.00	1.00	0.00	1.00	1.00	1.00	0.00	1.00	1.00	1.00	0.00
$F_{3}$(1D)	1.00	1.00	1.00	0.00	1.00	1.00	1.00	0.00	1.00	1.00	1.00	0.00
$F_{4}$(2D)	1.00	1.00	1.00	0.00	1.00	1.00	1.00	0.00	1.00	1.00	1.00	0.00
$F_{5}$(2D)	1.00	1.00	1.00	0.00	1.00	1.00	1.00	0.00	1.00	1.00	1.00	0.00
$F_{6}$(2D)	1.00	0.88	1.00	0.02	1.00	0.91	0.99	0.02	1.00	0.91	0.99	0.02
$F_{7}$(2D)	1.00	1.00	1.00	0.00	1.00	1.00	1.00	0.00	1.00	1.00	1.00	0.00
$F_{6}$(3D)	1.00	0.93	0.99	0.02	1.00	0.93	0.99	0.02	1.00	0.93	0.99	0.02
$F_{7}$(3D)	1.00	1.00	1.00	0.00	1.00	1.00	1.00	0.00	1.00	1.00	1.00	0.00
$F_{8}$(2D)	1.00	1.00	1.00	0.00	1.00	1.00	1.00	0.00	1.00	1.00	1.00	0.00
$F_{9}$(2D)	1.00	1.00	1.00	0.00	1.00	1.00	1.00	0.00	1.00	1.00	1.00	0.00
$F_{10}$(2D)	1.00	0.00	0.87	0.31	1.00	0.00	0.86	0.31	1.00	0.00	0.86	0.31
$F_{11}$(2D)	1.00	1.00	1.00	0.00	1.00	1.00	1.00	0.00	1.00	1.00	1.00	0.00
$F_{11}$(3D)	1.00	1.00	1.00	0.00	1.00	1.00	1.00	0.00	1.00	1.00	1.00	0.00
$F_{12}$(3D)	1.00	1.00	1.00	0.00	1.00	1.00	1.00	0.00	1.00	1.00	1.00	0.00
$F_{11}$(5D)	1.00	1.00	1.00	0.00	1.00	1.00	1.00	0.00	1.00	1.00	1.00	0.00
$F_{12}$(5D)	1.00	1.00	1.00	0.00	1.00	1.00	1.00	0.00	1.00	1.00	1.00	0.00
$F_{11}$(10D)	1.00	1.00	1.00	0.00	1.00	1.00	1.00	0.00	1.00	1.00	1.00	0.00
$F_{12}$(10D)	1.00	0.00	0.22	0.41	1.00	0.00	0.22	0.41	1.00	0.00	0.22	0.41
$F_{12}$(20D)	1.00	1.00	1.00	0.00	1.00	1.00	1.00	0.00	1.00	1.00	1.00	0.00

**Table 6:** Best, worst, average, and standard deviation of recall across 50 independent runs of NCDE.

Function	PI				HVPI				HVPIC			
	***Best***	***Worst***	***Avg.***	***Std.***	***Best***	***Worst***	***Avg.***	***Std.***	***Best***	***Worst***	***Avg.***	***Std.***
$F_{1}$(1D)	1.00	1.00	1.00	0.00	1.00	1.00	1.00	0.00	1.00	1.00	1.00	0.00
$F_{2}$(1D)	1.00	1.00	1.00	0.00	1.00	1.00	1.00	0.00	1.00	1.00	1.00	0.00
$F_{3}$(1D)	1.00	1.00	1.00	0.00	1.00	1.00	1.00	0.00	1.00	1.00	1.00	0.00
$F_{4}$(2D)	0.75	0.75	0.75	0.00	1.00	1.00	1.00	0.00	1.00	1.00	1.00	0.00
$F_{5}$(2D)	0.50	0.50	0.50	0.00	1.00	1.00	1.00	0.00	1.00	1.00	1.00	0.00
$F_{6}$(2D)	0.50	0.28	0.44	0.05	0.89	0.44	0.66	0.10	0.89	0.44	0.66	0.10
$F_{7}$(2D)	0.47	0.33	0.41	0.03	0.86	0.69	0.78	0.04	0.86	0.69	0.78	0.04
$F_{6}$(3D)	0.32	0.20	0.29	0.03	0.75	0.28	0.54	0.10	0.75	0.28	0.54	0.10
$F_{7}$(3D)	0.26	0.20	0.23	0.01	0.61	0.46	0.52	0.03	0.61	0.46	0.52	0.03
$F_{8}$(2D)	1.00	1.00	1.00	0.00	1.00	1.00	1.00	0.00	1.00	1.00	1.00	0.00
$F_{9}$(2D)	0.50	0.50	0.50	0.00	0.67	0.67	0.67	0.00	0.67	0.67	0.67	0.00
$F_{10}$(2D)	0.38	0.00	0.20	0.11	0.50	0.00	0.23	0.12	0.50	0.00	0.23	0.12
$F_{11}$(2D)	0.50	0.33	0.47	0.07	0.67	0.50	0.63	0.07	0.67	0.50	0.63	0.07
$F_{11}$(3D)	0.67	0.50	0.66	0.02	0.67	0.50	0.66	0.02	0.67	0.50	0.66	0.02
$F_{12}$(3D)	0.38	0.25	0.28	0.05	0.38	0.25	0.28	0.05	0.38	0.25	0.28	0.05
$F_{11}$(5D)	0.67	0.50	0.64	0.06	0.67	0.50	0.64	0.06	0.67	0.50	0.64	0.06
$F_{12}$(5D)	0.25	0.13	0.24	0.03	0.25	0.13	0.24	0.03	0.25	0.13	0.24	0.03
$F_{11}$(10D)	0.17	0.17	0.17	0.00	0.33	0.33	0.33	0.00	0.33	0.33	0.33	0.00
$F_{12}$(10D)	0.13	0.00	0.03	0.05	0.13	0.00	0.03	0.05	0.13	0.00	0.03	0.05
$F_{12}$(20D)	0.25	0.13	0.25	0.02	0.25	0.13	0.25	0.02	0.25	0.13	0.25	0.02

**Table 7:** Best, worst, average, and standard deviation of precision across 50 independent runs of LIPS.

Function	PI				HVPI				HVPIC			
	***Best***	***Worst***	***Avg.***	***Std.***	***Best***	***Worst***	***Avg.***	***Std.***	***Best***	***Worst***	***Avg.***	***Std.***
$F_{1}$(1D)	1.00	1.00	1.00	0.00	1.00	1.00	1.00	0.00	1.00	1.00	1.00	0.00
$F_{2}$(1D)	1.00	1.00	1.00	0.00	1.00	1.00	1.00	0.00	1.00	1.00	1.00	0.00
$F_{3}$(1D)	1.00	1.00	1.00	0.00	1.00	1.00	1.00	0.00	1.00	1.00	1.00	0.00
$F_{4}$(2D)	1.00	1.00	1.00	0.00	1.00	1.00	1.00	0.00	1.00	1.00	1.00	0.00
$F_{5}$(2D)	1.00	1.00	1.00	0.00	1.00	1.00	1.00	0.00	1.00	1.00	1.00	0.00
$F_{6}$(2D)	1.00	1.00	1.00	0.00	1.00	1.00	1.00	0.00	1.00	1.00	1.00	0.00
$F_{7}$(2D)	1.00	1.00	1.00	0.00	1.00	1.00	1.00	0.00	1.00	1.00	1.00	0.00
$F_{6}$(3D)	1.00	1.00	1.00	0.00	1.00	1.00	1.00	0.00	1.00	1.00	1.00	0.00
$F_{7}$(3D)	1.00	1.00	1.00	0.00	1.00	1.00	1.00	0.00	1.00	1.00	1.00	0.00
$F_{8}$(2D)	1.00	1.00	1.00	0.00	1.00	1.00	1.00	0.00	1.00	1.00	1.00	0.00
$F_{9}$(2D)	1.00	1.00	1.00	0.00	1.00	1.00	1.00	0.00	1.00	1.00	1.00	0.00
$F_{10}$(2D)	1.00	1.00	1.00	0.00	1.00	1.00	1.00	0.00	1.00	1.00	1.00	0.00
$F_{11}$(2D)	1.00	1.00	1.00	0.00	1.00	1.00	1.00	0.00	1.00	1.00	1.00	0.00
$F_{11}$(3D)	1.00	1.00	1.00	0.00	1.00	1.00	1.00	0.00	1.00	1.00	1.00	0.00
$F_{12}$(3D)	1.00	1.00	1.00	0.00	1.00	1.00	1.00	0.00	1.00	1.00	1.00	0.00
$F_{11}$(5D)	1.00	1.00	1.00	0.00	1.00	1.00	1.00	0.00	1.00	1.00	1.00	0.00
$F_{12}$(5D)	1.00	1.00	1.00	0.00	1.00	1.00	1.00	0.00	1.00	1.00	1.00	0.00
$F_{11}$(10D)	1.00	0.00	0.96	0.20	1.00	0.00	0.63	0.18	1.00	0.00	0.63	0.17
$F_{12}$(10D)	1.00	0.00	0.90	0.30	1.00	0.00	0.87	0.30	1.00	0.00	0.89	0.30
$F_{12}$(20D)	1.00	0.00	0.80	0.40	1.00	0.00	0.80	0.40	1.00	0.00	0.80	0.40

**Table 8:** Best, worst, average, and standard deviation of recall across 50 independent runs of LIPS.

Function	PI				HVPI				HVPIC			
	***Best***	***Worst***	***Avg.***	***Std.***	***Best***	***Worst***	***Avg.***	***Std.***	***Best***	***Worst***	***Avg.***	***Std.***
$F_{1}$(1D)	1.00	1.00	1.00	0.00	1.00	1.00	1.00	0.00	1.00	1.00	1.00	0.00
$F_{2}$(1D)	1.00	1.00	1.00	0.00	1.00	1.00	1.00	0.00	1.00	1.00	1.00	0.00
$F_{3}$(1D)	1.00	1.00	1.00	0.00	1.00	1.00	1.00	0.00	1.00	1.00	1.00	0.00
$F_{4}$(2D)	0.75	0.75	0.75	0.00	1.00	1.00	1.00	0.00	1.00	1.00	1.00	0.00
$F_{5}$(2D)	0.50	0.50	0.50	0.00	1.00	1.00	1.00	0.00	1.00	1.00	1.00	0.00
$F_{6}$(2D)	0.50	0.39	0.48	0.03	0.89	0.44	0.66	0.09	0.89	0.44	0.66	0.09
$F_{7}$(2D)	0.39	0.25	0.32	0.03	0.58	0.39	0.48	0.05	0.58	0.39	0.48	0.05
$F_{6}$(3D)	0.33	0.22	0.28	0.02	0.43	0.27	0.35	0.03	0.43	0.27	0.35	0.03
$F_{7}$(3D)	0.18	0.13	0.15	0.01	0.27	0.18	0.21	0.02	0.27	0.18	0.21	0.02
$F_{8}$(2D)	1.00	0.75	0.90	0.07	1.00	0.75	0.90	0.07	1.00	0.75	0.90	0.07
$F_{9}$(2D)	0.50	0.33	0.48	0.06	0.67	0.50	0.64	0.06	0.67	0.50	0.64	0.06
$F_{10}$(2D)	0.50	0.13	0.28	0.08	0.63	0.25	0.41	0.09	0.63	0.25	0.41	0.09
$F_{11}$(2D)	0.50	0.33	0.46	0.07	0.67	0.50	0.63	0.07	0.67	0.50	0.63	0.07
$F_{11}$(3D)	0.67	0.50	0.65	0.05	0.67	0.50	0.65	0.05	0.67	0.50	0.65	0.05
$F_{12}$(3D)	0.63	0.13	0.46	0.12	0.63	0.13	0.46	0.12	0.63	0.13	0.46	0.12
$F_{11}$(5D)	0.50	0.17	0.28	0.10	0.50	0.17	0.28	0.10	0.50	0.17	0.28	0.10
$F_{12}$(5D)	0.50	0.13	0.25	0.10	0.50	0.13	0.33	0.11	0.50	0.13	0.33	0.11
$F_{11}$(10D)	0.17	0.00	0.16	0.03	0.50	0.00	0.33	0.15	0.50	0.00	0.32	0.14
$F_{12}$(10D)	0.25	0.00	0.12	0.05	0.25	0.00	0.17	0.08	0.25	0.00	0.17	0.08
$F_{12}$(20D)	0.25	0.00	0.10	0.05	0.50	0.00	0.13	0.10	0.50	0.00	0.13	0.10

**Table 9:** Experimental results for $F_{7}$(3D) under different degrees of convergence.

MaxFEs		10,000		25,000		50,000		100,000	
**Alg.**		***Avg.***	***Std.***	***Avg.***	***Std.***	***Avg.***	***Std.***	***Avg.***	***Std.***
PI_0_	$|S_{\mathrm{OS}}\cap S_{\mathrm{GO}}|$	11.36	2.36	40.20	3.05	58.98	3.98	67.66	3.35
	*Precision*	0.02	0.00	0.08	0.01	0.12	0.01	0.14	0.01
	*Recall*	0.05	0.01	0.19	0.01	0.27	0.02	0.31	0.02
	*F*	$0.03_{b}^{*}$	0.01	$0.11_{b}^{*}$	0.01	$0.16_{b}^{*}$	0.01	$0.19_{b}^{*}$	0.01
PL	$|S_{\mathrm{OS}}|$	11.92	2.35	30.90	2.20	39.00	2.56	42.80	2.74
	$|S_{\mathrm{OS}}\cap S_{\mathrm{GO}}|$	10.90	2.08	30.90	2.20	39.00	2.56	42.80	2.74
	*Precision*	0.92	0.10	1.00	0.00	1.00	0.00	1.00	0.00
	*Recall*	0.05	0.01	0.14	0.01	0.18	0.01	0.20	0.01
	*F*	0.10	0.02	$0.25_{b}^{*}$	0.02	$0.31_{b}^{*}$	0.02	$0.33_{b}^{*}$	0.02
HVPI	$|S_{\mathrm{OS}}|$	12.58	2.68	40.22	3.07	58.98	3.98	67.66	3.35
	$|S_{\mathrm{OS}}\cap S_{\mathrm{GO}}|$	11.36	2.36	40.20	3.05	58.98	3.98	67.66	3.35
	*Precision*	0.91	0.11	1.00	0.00	1.00	0.00	1.00	0.00
	*Recall*	0.05	0.01	0.19	0.01	0.27	0.02	0.31	0.02
	*F*	0.10	0.02	0.31	0.02	0.43	0.02	0.48	0.02
	#FEs	174.18	56.28	3761.56	405.11	9210.18	578.32	11,439.86	572.74
HVPIC	$|S_{\mathrm{OS}}|$	12.58	2.68	40.22	3.07	59.00	3.99	67.66	3.35
	$|S_{\mathrm{OS}}\cap S_{\mathrm{GO}}|$	11.36	2.36	40.20	3.05	58.98	3.98	67.66	3.35
	*Precision*	0.91	0.11	1.00	0.00	1.00	0.00	1.00	0.00
	*Recall*	0.05	0.01	0.19	0.01	0.27	0.02	0.31	0.02
	*F*	0.10	0.02	0.31	0.02	0.43	0.02	0.48	0.02
	#FEs	35.14	8.87	188.16	15.66	300.70	17.26	355.16	15.32

The notation ‘*’ (‘b’) indicates that the *F* value achieved by HVPI (HVPIC) is significantly better than that of the corresponding algorithm according to the *t*-test at significance level 0.05.

**Table 10:** Experimental results for $F_{7}$(3D) under different accuracy levels.

Accuracy Level ε		0.1		0.01		0.001		0.0001	
**Alg.**		***Avg.***	***Std.***	***Avg.***	***Std.***	***Avg.***	***Std.***	***Avg.***	***Std.***
PI_0_	$|S_{\mathrm{OS}}\cap S_{\mathrm{GO}}|$	78.18	5.19	58.98	3.98	42.14	2.59	31.00	2.88
	*Precision*	0.16	0.01	0.12	0.01	0.08	0.01	0.06	0.01
	*Recall*	0.36	0.02	0.27	0.02	0.20	0.01	0.14	0.01
	*F*	$0.22_{b}^{*}$	0.01	$0.16_{b}^{*}$	0.01	$0.12_{b}^{*}$	0.01	$0.09_{b}^{*}$	0.01
PL	$|S_{\mathrm{OS}}|$	44.42	2.32	39.00	2.56	32.52	2.26	25.80	2.37
	$|S_{\mathrm{OS}}\cap S_{\mathrm{GO}}|$	44.38	2.36	39.00	2.56	32.52	2.26	25.80	2.37
	*Precision*	1.00	0.00	1.00	0.00	1.00	0.00	1.00	0.00
	*Recall*	0.21	0.01	0.18	0.01	0.15	0.01	0.12	0.01
	*F*	$0.34_{b}^{*}$	0.02	$0.31_{b}^{*}$	0.02	$0.26_{b}^{*}$	0.02	$0.21_{b}^{*}$	0.02
HVPI	$|S_{\mathrm{OS}}|$	78.18	5.19	58.98	3.98	42.14	2.59	31.00	2.88
	$|S_{\mathrm{OS}}\cap S_{\mathrm{GO}}|$	78.18	5.19	58.98	3.98	42.14	2.59	31.00	2.88
	*Precision*	1.00	0.00	1.00	0.00	1.00	0.00	1.00	0.00
	*Recall*	0.36	0.02	0.27	0.02	0.20	0.01	0.14	0.01
	*F*	0.53	0.03	0.43	0.02	0.33	0.02	0.25	0.02
	#FEs	12,145.56	668.27	9210.18	578.32	6158.72	593.62	3227.04	428.20
HVPIC	$|S_{\mathrm{OS}}|$	78.18	5.28	58.98	3.98	42.14	2.59	31.00	2.88
	$|S_{\mathrm{OS}}\cap S_{\mathrm{GO}}|$	77.86	5.20	58.98	3.98	42.14	2.59	31.00	2.88
	*Precision*	1.00	0.01	1.00	0.00	1.00	0.00	1.00	0.00
	*Recall*	0.36	0.02	0.27	0.02	0.20	0.01	0.14	0.01
	*F*	0.53	0.03	0.43	0.02	0.33	0.02	0.25	0.02
	#FEs	366.48	23.60	300.78	17.27	229.84	15.88	165.10	16.09

The notation ‘*’ (‘b’) indicates that the *F* value achieved by HVPI (HVPIC) is significantly better than that of the corresponding algorithm according to the *t*-test at significance level 0.05.

**Table 11:** Application of the proposed algorithm to engineering problems.

Problem	PL			HVPI			HVPIC		
	***Precision***	***Recall***	***F***	***Precision***	***Recall***	***F***	***Precision***	***Recall***	***F***
P1	1.00	0.53	0.69	1.00	0.94	0.97	1.00	0.94	0.97
P2	1.00	0.06	0.12	1.00	0.41	0.58	1.00	0.41	0.58
P3	1.00	0.30	0.46	1.00	0.53	0.69	1.00	0.53	0.69

## Data Availability

The data and software can be obtained upon request from the corresponding author.
